# Antioxidant and Pro-Oxidant Properties of Selected Clinically Applied Antibiotics: Therapeutic Insights

**DOI:** 10.3390/ph17101257

**Published:** 2024-09-24

**Authors:** Tibor Maliar, Marcela Blažková, Jaroslav Polák, Mária Maliarová, Eva Ürgeová, Jana Viskupičová

**Affiliations:** 1 Institute of Chemistry and Environmental Sciences, Faculty of Natural Sciences, University of Ss. Cyril and Methodius in Trnava, Nám. J. Herdu 2, 917 01 Trnava, Slovakia; maria.maliarova@ucm.sk; 2 Institute of Biology and Biotechnology, Faculty of Natural Sciences, University of Ss. Cyril and Methodius in Trnava, Nám. J. Herdu 2, 917 01 Trnava, Slovakia; marcela.blazkova@nppc.sk (M.B.); eva.urgeova@ucm.sk (E.Ü.); 3 National Agricultural and Food Centre, Hlohovecká 2, 951 41 Lužianky, Slovakia; 4 Helgeheim Inc., Palackého 6403, 911 01 Trenčín, Slovakia; jaroslav.polak@helgeheim.com; 5 Centre of Experimental Medicine SAS, Institute of Experimental Pharmacology and Toxicology, Slovak Academy of Sciences, Dúbravská cesta 9, 841 04 Bratislava, Slovakia; janaviskupicova@gmail.com

**Keywords:** oxidative stress, antioxidant activity, pro-oxidant activity, antibiotics

## Abstract

Background: The balance between antioxidants and pro-oxidants plays a significant role in the context of oxidative stress, influenced by both physiological and non-physiological factors. Objectives: In this study, 18 prescribed antibiotics (including doxycycline hydrochloride, tigecycline, rifampicin, tebipenem, cefuroxime, cefixime, potassium clavulanate, colistin, ampicillin, amoxicillin, amikacin, nalidixic acid, azithromycin, pipemidic acid trihydrate, pivmecillinam, aztreonam, fosfomycin sodium, and ciprofloxacin) were subjected to simultaneous determination of antioxidant and pro-oxidant potential to assess if pro-oxidant activity is a dominant co-mechanism of antibacterial activity or if any antibiotic exhibits a balanced effect. Methods: This study presents a recently developed approach for the simultaneous assessment of antioxidant and pro-oxidant potential on a single microplate in situ, applied to prescribed antibiotics. Results: Ten antibiotics from eighteen showed lower antioxidant or pro-oxidant potential, while five exhibited only mild potential with DPPH50 values over 0.5 mM. The pro-oxidant antioxidant balance index (PABI) was also calculated to determine whether antioxidant or pro-oxidant activity was dominant for each antibiotic. Surprisingly, three antibiotics—doxycycline hydrochloride, tigecycline, and rifampicin—showed significant measures of both antioxidant and pro-oxidant activities. Especially notable was tebipenem, a broad-spectrum, orally administered carbapenem, showed a positive PABI index ratio, indicating a dominant antioxidant over pro-oxidant effect. Conclusions: These findings could be significant for both therapy, where the antibacterial effect is enhanced by radical scavenging activity, and biotechnology, where substantial pro-oxidant activity might limit microbial viability in cultures and consequently affect yield.

## 1. Introduction

The anatomical therapeutic chemical (ATC) classification system currently records more than 200 antibiotics approved for clinical or veterinary use [1]. This relatively large number and diversity arise from the need for antibiotic therapy for different bacterial infections. The situation is further complicated by increasing bacterial resistance to available antibiotics [2,3]. Such infections were significant during the COVID-19 pandemic, where cytokine inflammatory storms [4] and secondary bacterial infections [5] significantly contributed to patient mortality. Managing these superinfections often involves combination antibiotic therapy [6]. The rise in bacterial resistance poses a significant threat, potentially leading to a shortage of effective antibiotics for future pandemics.

Antibiotics are widely recognized for their role in inhibiting bacterial growth by targeting vital cellular processes such as protein synthesis, nucleic acid replication, and cell wall biosynthesis [7]. Antibiotics can be classified as either bacteriostatic, halting bacterial growth and reproduction, or bactericidal, inducing oxidative stress within bacterial cells and leading to cell death [8]. Recent research has highlighted additional roles antibiotics may play in influencing the redox environment within bacterial and host cells. Specifically, antibiotics may exert dual pro-oxidant and antioxidant effects, contributing to their therapeutic efficacy or adverse effects. Pro-oxidant properties, often associated with oxidative stress generation, contribute to the bactericidal activity by inducing reactive oxygen species (ROS), reactive nitrogen species (RNS), or reactive carbonyl species, which damage bacterial DNA, proteins, and lipids, ultimately leading to cell death [9,10,11,12,13]. Several mechanisms have been described for ROS overproduction, such as the Krebs cycle and electron transport chain activation, leading to the formation of peroxide radicals [14]. Heleen van Acker and Tom Coenye proposed a mechanism for bacterial cell death due to hydroxyl radical production, which can be promoted by Fenton reactions involving transition metals like iron and copper [10]. Oxidative burst is a physiological mechanism by which macrophages eliminate bacterial cells [15]. The consequences of oxidative stress in bacterial cells include DNA breaks, lipid peroxidation, protein carbonylation, and other irreversible changes. ROS-induced cell death is primarily linked to the guanine nucleotide pool’s oxidative damage (the formation of 8-oxo-deoxyguanosine lesions), leading to oxidized nucleotides’ incorporation into RNA and DNA [12]. This ROS-mediated mechanism is often utilized by bactericidal antibiotics such as beta-lactams, fluoroquinolones, and aminoglycosides. Conversely, some antibiotics like minocycline, azithromycin, and doxycycline demonstrate antioxidant properties, which can mitigate unwanted oxidative damage in host tissues [16]. This balance between pro-oxidant and antioxidant effects may influence not only bacterial survival but also tissue recovery during infection. The dual properties of antibiotics—pro-oxidant and antioxidant—are of growing interest, as they may play a crucial role in therapeutic outcomes, resistance mechanisms, and even in biotechnological processes where antibiotics are used in microbial cultures. Antibiotics are primarily produced through the fermentation of soil microorganisms, particularly actinomycetes, micromycetes, and other groups of soil microbes. The predominant pro-oxidant effect of antibiotics can significantly negatively impact their biotechnological production. Penicillins, beta-lactam antibiotics, are originally derived from *Penicillium* fungi, such as *P. chrysogenum* and *P. rubens*, and are typically synthesized fermentatively [17]. Semisynthetic penicillins are derived from 6-aminopenicillinic acid (6-APA), which contains the beta-lactam core of Penicillin G but with modified side chains [18,19]. Broad-spectrum antibiotics (ampicillin and amoxicillin) are effective against a wide range of Gram-negative bacteria, including *Escherichia coli* and *Salmonella typhi*, and are further divided into carboxypenicillins and ureidopenicillins. Penicillins, for example, are beta-lactam antibiotics derived from Penicillium molds and are typically synthesized fermentatively. Tetracyclines and macrolides, such as erythromycin, are also produced fermentatively. The complex media used for these cultures contain nutrients and elements that can facilitate pro-oxidant effects through Fenton-like reactions. Similarly, cephalosporins are derived from 7-aminocephalosporanic acid (7-ACA), a core structure for synthesizing beta-lactam antibiotics originally isolated from *Cephalosporium acremonium* [20,21]. Fermentative production of tetracyclines served as a template for semisynthetic derivatives [22]. Chlortetracycline was first isolated in 1945 [23], followed by the structural identification of oxytetracycline from *Streptomyces rimosus* [24]. The first macrolide, erythromycin, was isolated in 1952 from *Saccharopolyspora erythraea* and is listed among the World Health Organization’s essential medicines. Antibiotics’ toxic effects on eukaryotic cells and their metabolic burden on the liver are significant [25]. Culture media are complex, containing essential nutrients and macro- and microelements. Numerous studies highlight the significant pro-oxidant effects of compounds with heteroatoms, particularly those with multiple bonds. These structural conformations can form coordination complexes with transition metals, contacting with hydrogen peroxide participating in Fenton and Fenton-like reactions to produce ROS and RNS [26,27,28].

Currently, various methods exist for measuring antioxidant and pro-oxidant potential, although there is no universally accepted definition or standard protocol for either. Common colorimetric assays for antioxidant activity determination include DPPH (2,2-diphenyl-1-picrylhydrazyl), TRAP (total radical-trapping antioxidant parameter), ABTS (2,2′-azino-bis(3-ethylbenzothiazoline-6-sulfonic acid)), TAR (total antioxidant response), ORAC (oxygen radical absorbance capacity), CUPRAC (cupric reducing antioxidant capacity), and FRAP (ferric reducing antioxidant power). Frequently used pro-oxidant activity measurement methods involve FRAP, Fenton reaction-based assays, TBARS (thiobarbituric acid reactive substances), and metal-catalyzed oxidation assays. The FRAP assay is particularly versatile, as it can be used to quantify both antioxidant and pro-oxidant activities by measuring substances that react with divalent or trivalent iron. In the context of pro-oxidant activity, the FRAP assay assesses the induction of lipid peroxidation. Another widely used method, the DPPH assay, measures antiradical activity but is limited to water-soluble antioxidants. These methods have inherent limitations and conventions, such as varying levels of specificity, sensitivity, and relevance, depending on the biological system and antioxidant or pro-oxidant properties being studied [29].

Recent advancements have allowed for the simultaneous determination of antioxidant and pro-oxidant potential using a single microplate to evaluate the pro-oxidant antioxidant balance index (PABI) [29]. Addressing the therapeutic aspects and biotechnological production of antibiotics is essential. Understanding the balance between antioxidant and pro-oxidant properties can enhance therapeutic efficacy by potentially improving infection outcomes and aiding tissue healing. Additionally, insights into pro-oxidant effects are crucial for optimizing biotechnological production processes, ensuring microbial viability and maximizing yield. Furthermore, this study could contribute to solving the problem of bacterial resistance by identifying antibiotic properties that enhance antibacterial efficacy through oxidative mechanisms, potentially leading to the development of more effective treatment strategies. Given the significance of oxidative stress in bacterial and host cell interactions, this study aims to further explore the antioxidant and pro-oxidant potential of 18 commonly prescribed antibiotics in relation to their physiological effects and the limitations of the biotechnological production of their precursors. Using the DPPH and FRAP assays, we investigate whether specific antibiotics favor pro-oxidant or antioxidant mechanisms under different conditions.

## 2. Results

We evaluated 18 selected antibiotics, each representing different mechanisms of action: doxycycline hydrochloride and tigecycline (tetracyclines) [30,31]; amikacin (aminoglycoside) [32] and azithromycin (macrolide) [33], which inhibit protein synthesis (PSI); beta-lactams, including penicillins (ampicillin, amoxicillin, and pivmecillinam) [34,35,36]; carbapenem (tebipenem) [37]; cephalosporins (cefuroxime and cefixime) [38,39]; monobactam (aztreonam) [40] and a beta-lactamase inhibitor (potassium clavulanate) [41], along with colistin (polymyxin E) [42] and fosfomycin sodium [43], which target the bacterial cell membrane and peptidoglycan in the cell wall (BCED); and rifampicin [44], nalidixic acid [45], pipemidic acid trihydrate [46], and ciprofloxacin [47], which act on bacterial DNA replication and transcription processes (NAB). The structures and CAS numbers of evaluated antibiotics presents Table S1 in supplementary file.

Antioxidant potential was determined using the DPPH method, and pro-oxidant potential was determined using the FRAP method, with results expressed as DPPH_50_ and FRAP_50_ values, respectively. The obtained results, along with antibiotic classification and antibacterial mechanisms, are presented in Table 1, ordered by descending antioxidant potential.

Only six antibiotics—doxycycline hydrochloride, tigecycline, rifampicin, tebipenem, cefuroxime, and cefixime—demonstrated detectable antioxidant potential. Potassium clavulanate exhibited nonsignificant pro-oxidant effect. Doxycycline hydrochloride (PABI index 0.27) and rifampicin (PABI index 0.26) showed significant pro-oxidant potential compared to their antioxidant properties, approximately fourfold, suggesting a link to their antibacterial mechanisms. In contrast, tebipenem, a carboxypenicillin, displayed an unexpectedly high antioxidant potential (PABI index = 6.12), indicating a strong radical scavenging ability that may contribute to the healing process in inflamed tissues near infection sites. All other antibiotics did not exhibit any detectable antioxidant or pro-oxidant potential within the tested concentration range up to 4096 μM, indicating that their antibacterial effects are likely based on mechanisms other than redox modulation.

**Figure 1:**
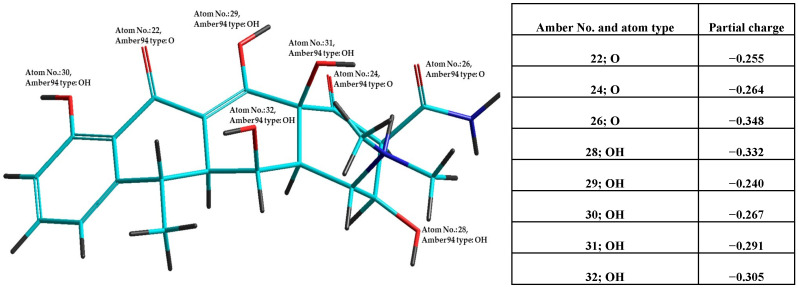
Structure of the antibiotic doxycycline quaternary cation after MM-QM optimization. Oxygen atoms are labeled with their atom number and type, and partial charges are provided in the accompanying table.

To elucidate the observed antioxidant and pro-oxidant potential, in silico calculations were conducted using the Molinspiration program/Molinspiration Cheminformatics 2024/, (Table 2). For all 18 selected antibiotics, parameters based on Lipinski’s rule of five were calculated, including the number of violations of these criteria and the bioactivity predictions across six different mechanisms. These in silico predictions are complemented by results from calculations using Hyperchem 7.5. For demonstration, Figure 1 presents the optimized structure of the antibiotic doxycycline hydrochloride after combined molecular mechanics (semiempirical quantum mechanics /MM-QM/ calculation, with oxygen atoms labeled and partial charges indicated).

Bioactivity predictions, expressed as dimensionless scores, are significant for positive values or those nearing the maximum of 0.5. While the primary mechanisms of action for antibiotics are well-established, such as the inhibition of penicillin-binding proteins (PBPs) by beta-lactams (penicillins, cephalosporins, carbapenems, and monobactams), alternative mechanisms also exist. Molinspiration’s predictions indicate various potential mechanisms. Six antibiotics, including ampicillin, amoxicillin, amikacin, pivmecillinam, and aztreonam, may act as GPCR ligands or inhibitors of proteases and enzymes. Cefuroxime and cefixime (cephalosporins) demonstrate potential as protease and enzyme inhibitors, as do tetracycline, doxycycline hydrochloride, and potassium clavulanate. Ciprofloxacin (a fluoroquinolone) also shows potential as a GPCR ligand and protease inhibitor.

Using Hyperchem software, we analyzed the oxygen atoms in antibiotic molecules. As heteroatoms, oxygen atoms are significant hydrogen bond acceptors and, as hydroxyl groups, also hydrogen bond donors. Hydroxyl groups on sp^2^ hybridized carbon atoms can participate in hydrogen atom transfer (HAT) mechanisms, with hydrogen bond formation influenced by the OH group’s hybridization and the partial charge on oxygen, affecting bond polarity. The data in Table 2 show that the number and partial charge of oxygen atoms are unique structural characteristics for each antibiotic. The antibiotics in both tables are ranked by descending antioxidant activity, correlated with the number of OH groups on sp2 hybridized carbon atoms (aromatic OH groups). These oxygen atoms exhibit lower partial charges, as reflected in the last two columns of the table. This is due to the +M effect of conjugating OH groups with aromatic rings, enhancing conditions for hydrogen bond donation via the HAT mechanism. The primary impact of antibiotics may be augmented by pro-oxidant effects and the formation of ROS/RNS in bacterial cells, as indicated by FRAP_50_ values. This is particularly evident for tetracyclines and Rifamycin, which exhibit antioxidant potential. Among tetracyclines, tigecycline shows a balanced antioxidant and pro-oxidant effect. Tebipenem, a carbapenem, demonstrates predominantly antioxidant potential, which, although not high, may contribute to the healing process of post-infection clearance.

**Figure 2:**
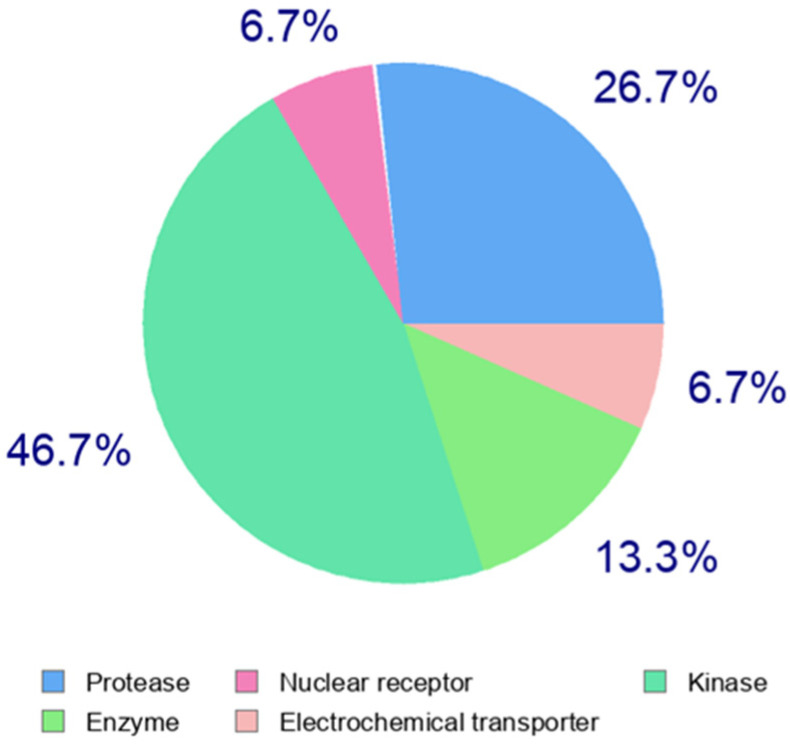
Target prediction analysis of possible human targets for the tetracycline doxycycline hydrochloride.

The effects of antibiotics may not be strictly monofactorial. The complete impact on the biochemical pathways of prokaryotic and eukaryotic cells is likely not fully understood. The diversity and variability of possible antibiotic effects, even among structurally similar compounds within the same group, are shown in Table 3. This table outlines the estimated potential targets (target categories) in human cells and systems based on the structure of the antibiotic. The analysis was processed using the Swiss target prediction web application, listing the 2–3 target categories with the highest probability of interaction, and specifying the top-predicted target in the last column. For illustration, Figure 2 presents a pie chart for the tetracycline doxycycline hydrochloride, demonstrating the high structural variability of antibiotics and the corresponding range of potential targets.

The differences are also evident when comparing pairs of similar antibiotics, such as doxycycline hydrochloride vs. tigecycline or ampicillin vs. amoxicillin. Generally, antibiotics target various biochemical pathways, primarily inhibiting kinases, lyases, oxidoreductases, phosphatases, and enzymes in general. They also engage G protein-coupled receptors (families A and E) and nuclear receptors. To a lesser extent, they affect electrochemical transporters, cytosolic proteins, and other targets. This multifactorial impact can be approximated to predict potential targets in prokaryotic cells as well. Additionally, this target prediction analysis is valuable for estimating the potential toxicity of antibiotics in the human body.

## 3. Discussion

Our study investigated the antioxidant and pro-oxidant properties of 18 clinically applied antibiotics, aiming to understand their dual roles in therapeutic efficacy and potential biotechnological implications. We tested 2 out of 18 tetracycline antibiotics, 2 out of 50 cephalosporin antibiotics, and 1 out of 23 macrolide antibiotics. The study revealed that several clinically relevant antibiotics exhibit both antioxidant and pro-oxidant potential, which may contribute to their therapeutic mechanisms and influence host responses during bacterial infections. The balance between these properties is crucial in determining whether an antibiotic primarily acts through oxidative stress induction or provides protective antioxidant effects to host tissues. These results highlight the complexity of antibiotic actions beyond their primary antibacterial mechanisms.

Our comprehensive in silico study (unpublished data) involving 214 clinically approved antibiotics confirmed a significant dominance of hydrogen bond acceptors over hydrogen bond donors. This finding suggests a prevailing pro-oxidant effect over the antioxidant effect. The data for a typical antibiotic show compounds of borderline size with marked polarity, as indicated by the following parameters: molecular weight (MW) = 464.93 g/mol, milogP = −0.217, number of atoms = 32, number of hydrogen bond acceptors = 10, number of hydrogen bond donors = 4, number of rotatable bonds = 6, and positive bioactivity predictions only in two cases: protease inhibitor (0.112) and general enzyme inhibitor (0.017).

Clinically approved antibiotics do not necessarily comply with all Lipinski criteria. For instance, tigecycline, rifamycin, colistin, and amikacin violate three out of five Lipinski criteria, often due to their molecular size, which affects molecular weight and the number of hydrogen bond donors and acceptors. Despite these violations, these antibiotics are approved, indicating that not all clinically approved drugs must strictly adhere to these criteria.

The observed antioxidant and pro-oxidant potential provide insights into the potential mechanisms through which antibiotics exert their therapeutic effects. The generation of ROS and subsequent oxidative damage are essential components of the bactericidal activity of antibiotics. Antibiotics that induce oxidative stress have been shown to cause significant physiological alterations in bacterial cells, contributing to their lethality [48,49]. Among the antibiotics tested, doxycycline hydrochloride and rifampicin exhibited significant pro-oxidant properties, suggesting that their antibacterial mechanisms likely involve oxidative stress induction. Both antibiotics displayed high ROS generation, likely contributing to bacterial DNA damage and cellular death. Kohanski et al. [50] demonstrated that bactericidal antibiotics, such as beta-lactams, quinolones, and aminoglycosides, stimulate the production of harmful hydroxyl radicals through an oxidative damage pathway. Interestingly, the antioxidant potential of these antibiotics was relatively low, indicating that their primary mode of action is through ROS-mediated mechanisms.

On the other hand, tebipenem showed a distinct antioxidant profile, with significantly higher antioxidant potential compared to its pro-oxidant effect. This finding aligns with previous reports of tebipenem’s role in reducing oxidative stress in host tissues, which may facilitate tissue recovery post-infection. The strong radical scavenging ability of Tebipenem could play a supportive role in clinical settings, particularly in inflammatory conditions where excessive ROS could exacerbate tissue damage. However, it is important to consider the risk of antioxidant stress, where an overabundance of antioxidants may suppress essential ROS signaling and disrupt redox balance. This phenomenon may also create an environment that is harmful to micro-organisms, as seen in studies where antiviral drugs with strong antioxidant properties effectively counteract viral oxidative stress. While a pro-oxidant effect is desirable for its bactericidal action, antioxidant properties, like those of tebipenem and tetracyclines, may not diminish antibacterial efficacy. Instead, they may help in post-infection tissue repair, but caution should be taken regarding the balance of redox regulation. This unique antioxidant property contrasts with the ROS generation typically observed in other beta-lactams and highlights the complexity of redox balance in antibiotic therapy. Furthermore, the data from the DPPH and FRAP assays revealed that the majority of antibiotics tested did not exhibit significant redox-modulating effects at the concentrations studied. This suggests that the primary mechanisms of action for most antibiotics are likely unrelated to redox balance and are instead driven by direct inhibition of bacterial cellular processes. However, understanding the redox properties of antibiotics like tebipenem and rifampicin could inform future therapeutic strategies, particularly in cases where managing oxidative stress is crucial to patient recovery.

This mechanism, in which oxidative stress and resulting genomic instability play a central role in the effectiveness of antibacterial agents, highlights the complexity of antibiotic action [51]. The published pro-oxidant effects of nitrofurantoin [52] and polymyxin B [53], although not included in our test collection, further support this mechanism. Our study did not observe significant oxidative or pro-oxidant effects for colistin (polymyxin E). Hoeksema et al. [54] confirmed that ROS production is an additional mechanism of action for beta-lactam antibiotics, quinolones, and aminoglycosides, with interesting implications for de novo-acquired resistance. Under aerobic conditions, beta-lactam antibiotics at higher concentrations trigger significant ROS production in *Enterococcus faecalis*, whereas this is not observed under anaerobic conditions, suggesting an interaction mechanism with the respiratory chain [55].

Besides antibiotics, ROS generation is typical for metallic nanoparticles as green synthesis products [56]. They generate high amounts of ROS which cause damage to bacterial cells. Nanoparticles made of silver, silver oxide, titanium dioxide, silicon, copper oxide, zinc oxide, gold, calcium oxide, and magnesium oxide have been studied and found to be effective against both Gram-positive and Gram-negative bacteria [57].

It is essential to distinguish between direct ROS production, observed in the presence of transition metals by the antibiotics identified in our study, and indirect ROS production as a terminal state of blocked key metabolic pathways in bacterial cells. In this context, attention should be given to antibiotics that inhibit oxidoreductases or electrochemical transporters, as indicated in Table 3.

The findings of this study have significant implications for the biotechnological production of antibiotics. Understanding the balance between antioxidant and pro-oxidant properties can aid in optimizing fermentation processes and improving the yield and stability of antibiotic production. Antibiotics that exhibit high pro-oxidant activity may pose challenges during production, as oxidative stress can negatively affect the microbial strains used in fermentation. Conversely, antibiotics with notable antioxidant properties, such as tebipenem, may enhance microbial resilience and improve overall production efficiency. Future biotechnological strategies could involve modifying culture conditions to mitigate oxidative stress or engineering microbial strains with enhanced antioxidant defenses to boost production yields [58]. By considering the redox properties of antibiotics, biotechnological processes can be tailored to maximize productivity while ensuring the quality and efficacy of the final product.

### Limitations

The limitations of the DPPH and FRAP assays have been well documented in the literature. Both methods utilize synthetic reagents that do not fully mimic physiological conditions, which restricts their ability to represent in vivo antioxidant or pro-oxidant dynamics accurately. The DPPH assay, which measures the ability of compounds to scavenge the stable DPPH radical, primarily reflects the interaction with a model radical that does not exist in biological systems. This reduces the physiological relevance of the assay when interpreting how antioxidants behave in complex biological environments.

## 4. Materials and Methods

### 4.1. Materials and Chemicals

Chemicals and solvents for assays, such as 2,2-diphenyl-1-picrylhydrazyl radical (DPPH^●^), 2,2-diphenyl-1-picrylhydrazine (DPPH), 2,4,6-tris(2-pyridyl)-s-triazine (TPTZ), FeCl_2_·4H_2_O, FeCl_3_·6H_2_O, TROLOX, sodium acetate, ethanol, and acetic acid were purchased from Merck/Sigma (St. Louis, MO, USA). Similarly, the following antibiotics were purchased as standards from Merck/Sigma (USA): doxycycline hydrochloride, tigecycline, rifampicin, tebipenem, cefuroxime, cefixime, potassium clavulanate, colistin, ampicillin, amoxicillin, amikacin, nalidixic acid, azithromycin, pipemidic acid trihydrate, pivmecillinam, aztreonam, fosfomycin sodium, and ciprofloxacin. Standard 96-microwell plates (F-type, SPL Life Sciences, Pocheon-si, Republic of Korea) were used.

### 4.2. Measurement of Antioxidant and Pro-Oxidant Properties Using the Modified DPPH/FRAP Method

The modified DPPH/FRAP method was used to simultaneously measure the antioxidant and pro-oxidant properties of compounds, as described by Maliar et al. [29]. The DPPH method was used to measure antioxidant activity, and the FRAP method was used to measure both antioxidant and pro-oxidant activities. Modified microplate assays ensured equal concentrations of key reagents (DPPH, TPTZ, FeCl_3_) at 0.4 mM. Conversion standards were set with DPPH and FeCl_2_·4H_2_O for 100% conversion and DPPH^●^ and FeCl_3_·6H_2_O for 0% conversion.

The antibiotic solutions were diluted to achieve final concentrations ranging from 4096 μM to 8 μM. The microplate preparation involved applying conversion standards and an FeCl_3_ solution. Assays started with adding 0.4 mM DPPH and FRAP reagents, achieving a final concentration of 0.3 mM. The microplate was incubated for 10 min for DPPH and 1 h for FRAP, followed by measurements at 520 nm and 630 nm for DPPH and FRAP, respectively.

DPPH_50_ and FRAP_50_ values were calculated, with the pro-oxidant antioxidant balance index (PABI), determined as FRAP_50_/DPPH_50_ [28]. Each experiment was repeated three times with eight replicates, with results presented as mean ± SD. Statistical significance was determined using the Spearman method (* *p* < 0.1).

### 4.3. Molinspiration Calculation

The Molinspiration/Molinspiration Cheminformatics 2024/ calculations include Lipinski’s “drug-likeness” parameters (rule of five), based on the following:

- Octanol/water partition coefficient (LogP): calculated as a sum of fragment-based contributions and correction factors;
- Topological polar surface area (TPSA): calculated based on methodology of (https://molinspiration.com/ accessed on 17 September 2024) with summing fragment contributions fitted to the 3D volume of a training set of about 12,000, mostly drug-like, molecules. These geometries were optimized using the semi-empirical AM1 method;
- Rule of five: Most “drug-like” molecules have logP ≤ 5, molecular weight ≤ 500, ≤10 hydrogen bond acceptors, and ≤5 hydrogen bond donors;
- Number of rotatable bonds (nrotb): measures molecular flexibility and is a good descriptor of oral bioavailability and defined as any single non-ring bond bound to a non-terminal heavy atom, excluding amide C-N bonds due to their high rotational energy barrier.

The number of violations of Lipinski’s “drug-likeness” parameters, specifying which parameters are outside the criteria, was recorded. In the second step, the bioactivity score predictions were calculated using the Molinspiration virtual screening toolkit (miscreen) for GPCR ligands (GPCRL), ion channel modulators (ICHM), kinase inhibitors (KI), nuclear receptor ligands (NRL), protease inhibitors (PI), and other enzyme inhibitors (EI). Finally, the average bioactivity scores across these six different mechanisms were calculated, as the exact mechanism of action in bacterial cells may not be fully known or described.

### 4.4. Swiss Target Prediction Calculation

SwissTargetPrediction (http://swisstargetprediction.ch/index.php, accessed on 17 September 2024) predicts bioactive molecule targets using 2D and 3D similarity measures with known ligands. Predictions are available for five organisms, with homology mapping for close paralogs and orthologs. The prediction utilizes a library of 370,000 known actives on over 3000 proteins across 3 species, sourced from the ChEMBL database. Similarity thresholds are 0.85 for ES5D (shape) and 0.65 for FP2 (2D). Below these thresholds, the known actives are not considered similar enough to be displayed. More details can be found at SwissTargetPrediction [29].

In this study, we applied the prediction of human targets. The structures of antibiotics were drawn by specifying SMILES in the designated text box.

### 4.5. Optimization of Antibiotics Structure in Vacuum for Parameters Calculation

The partial charges of key atoms (oxygens) in all evaluated antibiotic structures were calculated by Hyperchem 8.52 using a combination of molecular mechanics and semiempirical methods, shortly MM-QM optimization, (forcefield AMBER and AM1) under defined terminal conditions in Hyperchem 7.5 (Hypercube, St Gainesville, USA). The number of aromatic and non-aromatic oxygen atoms with their partial charges was recorded.

### 4.6. Statistical Evaluation

Each experiment was repeated three times with eight replicates. The results were presented as the mean ± standard deviation (SD). The correlation coefficient was calculated using the Spearman method. The statistical significance between groups was evaluated using the Student’s *t*-test, with a difference considered statistically significant when *p* < 0.1. Each experiment was repeated three times with eight replicates.

## 5. Conclusions

Among the 18 clinically applied antibiotics studied, only six demonstrated detectable antioxidant potential. Doxycycline hydrochloride and rifampicin exhibited significantly higher pro-oxidant effects compared to their antioxidant effects, suggesting a contribution to their antibacterial mechanisms. Tebipenem showed notable antioxidant properties, potentially aiding in tissue healing. Most antibiotics did not display significant redox potential, indicating that their antibacterial effects are likely based on mechanisms other than redox modulation. In silico analysis revealed that not all antibiotics adhere to Lipinski’s rule of five, highlighting exceptions in drug design. The position of oxygen atoms on the antibiotic molecule, along with their partial charges, significantly influences the molecule’s antioxidant and pro-oxidant properties. Understanding these mechanisms not only underscores the complexity of antibiotic action but also highlights potential targets for enhancing antibacterial efficacy and addressing bacterial resistance. This study’s findings provide valuable insights into the dual antioxidant and pro-oxidant properties of antibiotics, with implications for therapy and biotechnology. Future research should expand the collection of tested antibiotics for both antioxidant and pro-oxidant effects to proportionally represent each antibiotic group, aiming to achieve a better understanding of these properties and their impact on antibacterial action. Additionally, we plan to study radical production mechanisms in bacterial cultures exposed to homogeneous parameters or using EPR methods.

## Figures and Tables

**Table 1:** Antioxidant and pro-oxidant potential of selected antibiotics with their classification and antibacterial mechanisms.

Antibiotic	Antibiotic Category/ Mechanism	DPPH_50_ (μM)	r^2^	FRAP_50_ (μM)	r^2^	PABI
Doxycycline hydrochloride	TC’s/ PSI	22.3 ± 0.6	0.992	5.9 ± 0.4	0.941	0.27
Tigecycline	TCs/ PSI	88.1 ± 2.9	0.962	91.6 ± 2.7	0.936	1.04
Rifampicin	ANM’s/ NAB	129 ± 6.8	0.989	33.1 ± 2.2	0.983	0.26
Tebipenem	BLK’s/ BCED	520.1 ± 40.6	0.988	3182.2 ± 69.87	0.923	6.12
Cefuroxime	BLK’s/ BCED	1533 ± 120	0.951	1919 ± 39	0.936	1.25
Cefixime	BLK’s/ BCED	2762 ± 220	0.934	3294 ± 46	0.955	1.19
Clavulanate	BLK’s/ BCED	ND	N/A	2625 ± 61	0.998	ND
Colistin	P’s/ BCED	ND	N/A	ND	N/A	ND
Ampicillin	BLK’s/ BCED	ND	N/A	ND	N/A	ND
Amoxicillin	BLK’s/ BCED	ND	N/A	ND	N/A	ND
Amikacin	AG’s/ PSI	ND	N/A	ND	N/A	ND
Nalidixic acid	Q’s/ NAB	ND	N/A	ND	N/A	ND
Azithromycin	ML’s/ PSI	ND	N/A	ND	N/A	ND
Pipemidic acid trihydrate	PP’s/ NAB	ND	N/A	ND	N/A	ND
Pivmecillinam	BLK’s/ BCED	ND	N/A	ND	N/A	ND
Aztreonam	BLK’s/ BCED	ND	N/A	ND	N/A	ND
Fosfomycin sodium	PHA’s/ BCED	ND	N/A	ND	N/A	ND
Ciprofloxacin	FQ’s	ND	N/A	ND	N/A	ND

PABI—pro-oxidant antioxidant balance index, TC’s -tetracyclines, ANM’s –ansamycins, BLK’s—betalactams, P’s –polymyxines, AG’s—aminoglycosides, Q’s –quinolones, ML’s—Macrolides, PP’s—pyridopirimidines, PHA’s—fosfonic acids, FQ’s—fluoroquinolones, PSI—protein synthesis inhibition, NAB—nucleic acid blockers, BCED—bacterial cell envelope destroyers, ND—not detectable, N/A—not applicable.

**Table 2:** The calculations conducted by Molinspiration/Molinspiration Cheminformatics 2024/ and Hyperchem 7.5 software in silico.

Antibiotic	*Molinspiration Calculations*		*Hyperchem Calculations*			
	nVIOL *	AVG_Bioact **	nOat	nSP2Oat	∑p. ch.	AVG_p.ch.
Doxycycline hydrochloride	1 (nDHB)	−0.115 (PI; EI)	8	2	−2.59	−0.288
Tigecycline	3 (MW, nDHB, nAHV)	−0.21 (EI)	8	3	−2.42	−0.302
Rifampicin	3 (MW, nDHB, nAHB)	−2.11	13	3	−3.59	−0.299
Tebipenem	0	−0.04 (PI; EI)	6	0	−1.80	−0.300
Cefuroxim	1 (nAHB)	−0.28 (PI; EI)	8	0	−1.56	−0.222
Cefixim	1 (AHB)	−0.23 (PI; EI)	7	0	1.29	−0.284
Potassium clavulanate	0	−0.48 (PI; EI)	5	0	−1.37	−0.273
Colistin	3 (MW, nDHB, nAHB)	−3.8	13	0	−4.41	−0.339
Ampicilín	0	0.04 (GPCRL; PI; EI)	4	0	−1.40	−0.349
Amoxicilín	0	0.07 (GPCRL; PI; EI)	5	0	−1.67	−0.335
Amikacin	3 (MW, nDHB, nAHB)	0.33 (GPCRL; PI; EI)	13	0	−4.19	−0.323
Nalidixic acid	0	−0.16 (EI)	3	0	−0.95	−0.317
Azithromycin	2 (MW, nAHB)	−0.59	12	0	−3.4	−0.309
Pipemidic acid trihydrate	0	0.22 (GPCRL; KI; EI)	3	0	−0.95	−0.316
Pivmecillinam	1 (nRB)	0.08 (GPCRL; PI; EI)	5	0	−1.47	−0.295
Aztreonam	1 (nAHB)	0.08 (GPCRL; PI; EI)	8	0	−3.5	−0.438
Fosfomycine sodium	0	−2.6	4	0	−2.23	−0.557
Ciprofloxacin	0	0.12 (GPCRL; EI)	3	0	−0.95	−0.318

* nVIOL = number of violations of Lipinski’s criteria with detailed indication of the specific parameter that is outside the defined range. MW = molecular weight, nABH = number of acceptors of H bonds, nDHB = number of donors of H bonds, nRB = number of ratable bonds; ** AVG_Bioact.—Average of value of predicted bioactivity score, GPCRL—GPCR ligand, ICHM-ion channel modulator, KI-kinase inhibitor, NRL_nuclear receptor ligand, PI_ protease inhibitor, EI-enzyme inhibitor, the mechanisms with positive score values are mentioned. nOat—number of oxygen atoms, nSP2Oat—number of oxygen atoms bound to sp2 hybridized carbon, ∑p. ch.—sum of partial charges on oxygen atoms, AVG_p.ch.—average value of partial charges on oxygen atoms.

**Table 3:** Predicted human targets for selected antibiotics based on structural analysis.

Antibiotic	Target 1 (Probability)	Target 2 (Probability)	Target 3 (Probability)	Target with Highest Probability
Doxycycline hydrochloride	Kinases (46.7%)	Proteases (26.7%)	Enzymes in general (13.3%)	Matrix metalloproteinase 2
Tigecycline	G coupled receptor, family E (46.7%)	Kinases (26.7%)	Protease (26.7%)	G protein-coupled receptor kinase 6
Rifampicin	Kinases (46.7%)	Proteases (6.7%)	Enzymes in general (6.7%)	Bile salt export pump
Cefuroxime	Enzymes in general (26.7%)	Lyases (26.7%)	G coupled receptor, family A (46.7%)	PI3-kinase p110-gamma subunit
Cefixime	Enzymes in general (33%)	Kinases 20%	Proteases 13.3%	Dihydrofolate reductase
Potassium clavulanate	Enzymes in general (53.3%)	Proteases (26.7%)	Oxidoreductases (6.7%)	Leukocyte elastase
Colistin	Proteases (60%)	Kinases (6.7%)	Membrane receptors (6.7%)	Pepsinogen C
Ampicillin	Kinases (33%)	Proteases (26.7%)	Lyases (6.7%)	Integrin alpha-4/beta-1
Amoxicillin	G coupled receptor, Family A (20%)	Adhesion (20%)	Lyases (6.7%)	Integrin alpha-4/beta-1
Amikacin	G coupled receptor, Family A (33.3%)	Adhesion (20%)	Enzymes in general (6.7%)	Galectin-4
Nalidixic acid	Erasers (26.7%)	Electrochemical transporter (13.3%)	Kinases (13.3%)	Serotonin transporter
Azithromycin	G coupled receptor, Family A (26.7%)	Enzymes in general (13.3%)	Electrochemical transporter (13.3%)	Human Ether-a-go-go-related Gene
Pipemidic acid trihydrate	Kinases (26.7%)	Enzymes in general (26.7%)	Proteases (13.3%)	Autotaxin
Pivmecillinam	Kinases (33.3%)	G coupled receptor, Family A (26.7%)	Enzymes in general (20%)	Phosphodiesterase 7A
Aztreonam	G coupled receptor, Family A (46.7%)	Enzymes in general (26.7%)	Proteases in general (13.3%)	Hypoxia-inducible factor prolyl 4-hydroxylase
Fosfomycin sodium	Family A, G coupled receptor, (50%)	Transferases (50%)	-	GABA-B receptor
Ciprofloxacin	Enzymes in general (33.3%)	Enzymes in general (26.7%)	Kinases (13.3%)	Glycogen synthase kinase-3 beta

## Data Availability

The original contributions presented in the study are included in the article; further inquiries can be directed to the corresponding author.

## Supplementary Materials

The following supporting information can be downloaded at: https://www.mdpi.com/article/10.3390/ph17101257/s1, Table S1: The structures and CAS numbers of evaluated antibiotics.
