# Supplementary material for: Antioxidant and Pro-Oxidant Properties of Selected Clinically Applied Antibiotics: Therapeutic Insights
Source: Pharmaceuticals (Basel). 2024 Sep 24;17(10):1257. doi: 10.3390/ph17101257 (PMC11510234; doi:10.3390/ph17101257)
Supplement: Supplementary file 1 [file pharmaceuticals-17-01257-s001.zip › pharmaceuticals-3195529-supplementary.pdf]

Antibiotic	CAS Number	Structure
Doxycycline hydrochloride	564-25-0	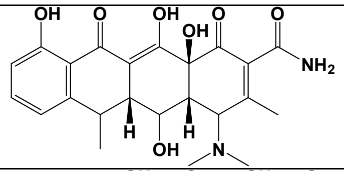
Tigecycline	220620-09-7	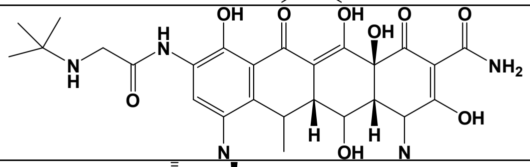
Rifampicin	13292-46-1	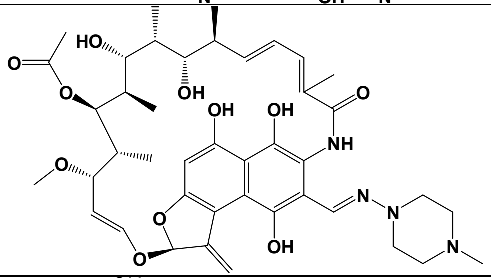
Tebipenem	161715-24-8	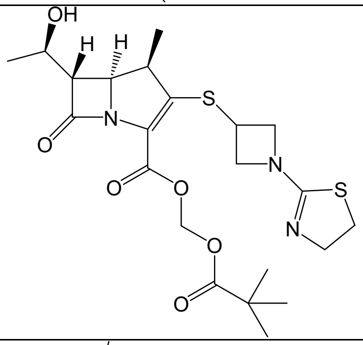
Cefuroxime	55268-75-2	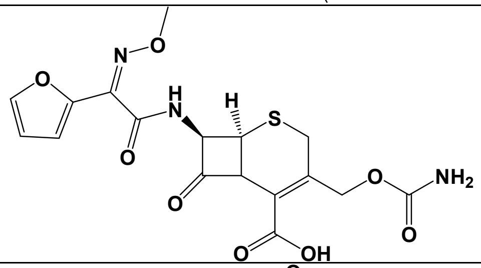
Cefixime	79350-37-1	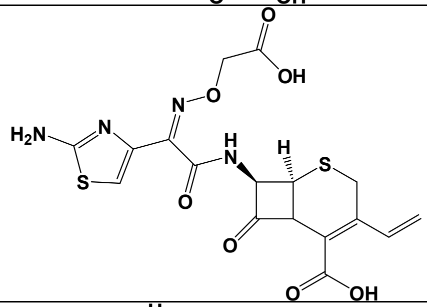
Clavulanate	58001-44-8	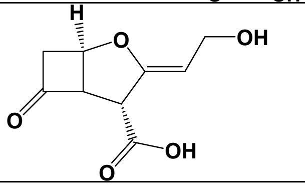

Colistin	1066-17-7	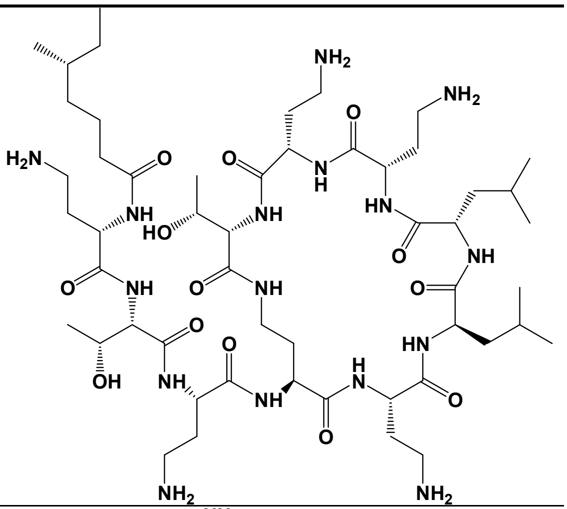
Ampicillin	69-53-4	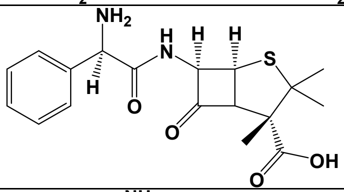
Amoxicillin	26787-78-0	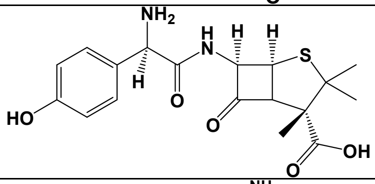
Amikacin	37517-28-5	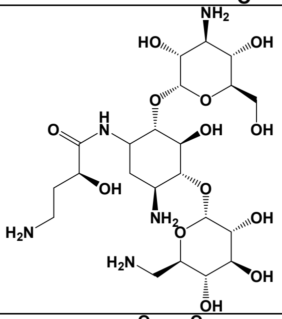
Nalidixic acid	389-08-2	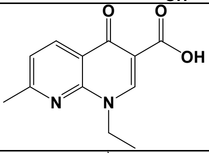
Azithromycin	83905-01-5	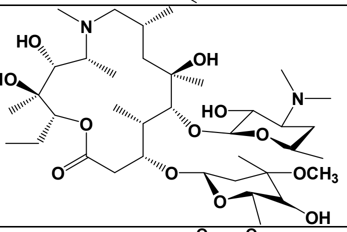
Pipemidic acid	51940-44-4	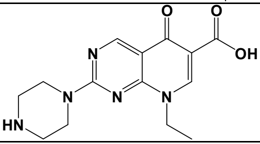

Pivmecillinam	32886-97-8	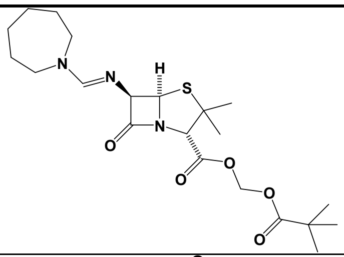
Aztreonam	78110-38-0	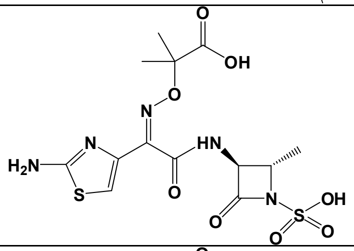
Fosfomycin sodium	23155-02-4	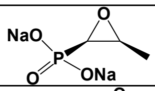
Ciprofloxacin	85721-33-1	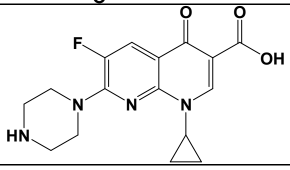
